# Multi-Atlas MRI-Based Striatum Segmentation for ^123^I-FP-CIT SPECT (DAT-SPECT) Compared With the Bolt Method and SPECT-Atlas-Based Segmentation Method Toward the Accurate Diagnosis of Parkinson's Disease/Syndrome

**DOI:** 10.3389/fmed.2021.662233

**Published:** 2021-05-25

**Authors:** Koji Sohara, Tetsuro Sekine, Amane Tateno, Sunao Mizumura, Masaya Suda, Takeshi Sakayori, Yoshiro Okubo, Shin-ichiro Kumita

**Affiliations:** ^1^Department of Radiology, Nippon Medical School Hospital, Tokyo, Japan; ^2^Department of Radiology, Nippon Medical School Musashi Kosugi Hospital, Kanagawa, Japan; ^3^Department of Neuropsychiatry, Nippon Medical School, Tokyo, Japan; ^4^Department of Radiology, Omori Medical Center, Toho University, Tokyo, Japan

**Keywords:** ^123^I-FP-CIT, ^18^F-FE-PE2I, positron emission tomography, semi-quantification, multi-atlas MRI based parcellation, Bolt method, single photon emission computed tomography, dopamine transporter

## Abstract

**Aims:** This study aimed to analyze the performance of multi-atlas MRI-based parcellation for ^123^I-FP-CIT SPECT (DAT-SPECT) in healthy volunteers. The proposed method was compared with the SPECT-atlas-based and Bolt methods. ^18^F-FE-PE2I-PET (DAT-PET) was used as a reference.

**Methods:** Thirty healthy subjects underwent DAT-SPECT, DAT-PET, and 3D-T1WI-MRI. We calculated the striatum uptake ratio (SUR/SBR), caudate uptake ratio (CUR), and putamen uptake ratio (PUR) for DAT-SPECT using the multi-atlas MRI-based method, SPECT-atlas-based method, and Bolt method. In the multi-atlas MRI-based method, the cerebellum, occipital cortex, and whole-brain were used as reference regions. The correlation of age with DAT-SPECT activity and the correlations of SUR/SBR, CUR, and PUR between DAT-SPECT and DAT-PET were calculated by each of the three methods.

**Results:** The correlation between age and SUR/SBR for DAT-SPECT based on the multi-atlas MRI-based method was comparable to that based on the SPECT-atlas-based method (*r* = −0.441 to −0.496 vs. −0.488). The highest correlation between DAT-SPECT and DAT-PET was observed using the multi-atlas MRI-based method with the occipital lobe defined as the reference region compared with the SPECT-atlas-based and Bolt methods (SUR, CUR, and PUR: 0.687, 0.723, and 0.676 vs. 0.698, 0.660, and 0.616 vs. 0.655).

**Conclusion:** Multi-atlas MRI-based parcellation with the occipital lobe defined as the reference region was at least comparable to the clinical methods.

## Introduction

^123^I-FP-CIT-SPECT (DAT-SPECT) is widely used for the assessment of degenerative Parkinson's syndromes, such as Parkinson's disease (PD), multiple system atrophy, and progressive supranuclear palsy. The tracer accumulation in DAT-SPECT reflects the availability of dopamine transporter (DAT) and thus the functionality of the nigrostriatal dopaminergic neurons (1–4). As a semi-quantitative method for DAT-SPECT, the uptake ratios in the corpus striatum or in more detailed regions, such as putamen and caudate nucleus, have been used. These ratios can be obtained by dividing the uptake in the target regions (e.g., corpus striatum) by those in the reference areas (e.g., cerebellum, occipital cortex, or whole brain). Several approaches have been attempted to provide robust semi-quantification from DAT imaging. In clinical settings, two types of methods, the Bolt method and the SPECT atlas method, are widely used. The Bolt method can serve as a robust, easy-to-use semi-quantification method (5). However, some errors are inevitable in the case of abnormal brain shapes (6). In addition, this approach does not segment the caudate and putamen, resulting in no capability to evaluate the distribution of nigrostriatal dopaminergic neuron changes in the striatum. Compared with the Bolt method, the SPECT atlas method (e.g., DaTQUANT, GE Healthcare, Little Chalfont, UK) can segment the caudate and putamen (7, 8). However, the segmentation may not be accurate because single SPECT template is used. The volume and shape of the striatum vary among subjects. Generally, it is observed that significant volume reduction is proportional to aging (9). One previous study demonstrated that the effect of aging on dopamine receptor availability was overestimated due to the volume reduction (10). To overcome the disadvantages of the current methods described above, Perlaki et al. evaluated the utility of segmentation by using patient-specific T1WI (11). Their method is expected to be easily implemented into clinical workflows because 3D-T1WI-MRI is generally performed as a routine assessment for Parkinson's diseases and the scan time for it has decreased due to recently developed acceleration techniques (e.g., parallel imaging and compressed sensing). However, they did not compare MRI segmentation to current clinical methods (the Bolt method or SPECT-atlas-based method). In addition, in terms of MRI segmentation, they only used single MRI atlas. One can expect that the MRI segmentation using multi-atlas MRI can improve the accuracy of segmentation (12).

The purpose of this study was to analyze the performance of multi-atlas MRI-based parcellation for DAT-SPECT. The proposed method was compared with the Bolt method and SPECT-atlas-based method, both of which are currently used in clinical settings.

^18^F-FE-PE2I ([18F]-(E)-N-(3-iodoprop-2-enyl)-2β-carbofluoroethoxy-3β-(4′-methyl-phenyl) nortropane) for PET (DAT-PET) was used as a reference. ^18^F-FE-PE2I showed high affinity and high selectivity for DAT, faster kinetics, more favorable metbolism and low production of a radio metabolite with intermediate lipophilicity (13–15). Several studies have confirmed its high correlation to age even in small regions such as caudate and putamen (*r* = −0.845 and −0.85, respectively) and high discriminative power between PD and healthy controls (16–19). Based on these previous reports, we expected that ^18^F-FE-PE2I was an excellent imaging tool for *in vivo* DAT quantification in the entire nigrostriatal tract (17, 18, 20).

## Materials and Methods

### Subjects

The study was approved by the institutional review board of our institution. Thirty healthy volunteers (HVs) aged 31–79 years (mean ± SD, 54.1 ± 14.5; six subjects per age group: 30s, 40s, 50s, 60s, and 70s) were enrolled. All studies were performed between January 2016 and March 2017. None of the subjects had a present or past history of psychiatric, neurological or somatic disorders or of alcohol- or drug-related problems. All subjects were non-smokers. After an explanation of the study, written informed consent was obtained from all participants. All participants underwent ^123^I-FP-CIT-SPECT (DAT-SPECT), ^18^F-PE2I-PET (DAT-PET) and 3D-T1WI-MRI within 85 days (21.4 ± 13.2 days).

### SPECT Procedures

SPECT imaging was performed using a dual-head SPECT gamma camera (Infinia, GE Healthcare, Milwaukee, WI, USA) equipped with an extended low-energy general-purpose (ELEGP) collimator (12 mm full width at half maximum; FWHM). All subjects had received an intravenous injection of ^123^I-FP-CIT (167 MBq) (^123^I-Ioflupane, DaTScan™, Nihon Mediphysics Corporation, Nishinomiya, Japan) *via* the antecubital vein in the supine position. Three hours after tracer injection, SPECT data were acquired for 28.5 min over a 360° range in 4°-angular steps (90 views) with 855 s/cycle using circularly rotating gamma cameras. The radius of rotation was 14 cm. We used a 3-dimensional ordered subset expectation maximization (3D-OSEM) image reconstruction algorithm (iteration number, 6; subsets, 10) with an eighth-order Butterworth filter with a cut off of 0.55 cycles/cm. The final SPECT images were reconstructed with Chang's method (Chang's attenuation correction) into 3.0-mm isotropic voxels using a 128 × 128 matrix with 128 slices parallel to the orbitomeatal line (21, 22). Scatter correction was not used in this study.

### PET Procedures

^18^F-FE-PE2I was synthesized from its precursor, tosylethyl-PE2I, *via* a nucleophilic fluorination reaction in our cyclotron for PET (HM18, Sumitomo Heavy Industries, Ltd, Tokyo) at the Clinical Imaging Center for Healthcare, Nippon Medical School. PET scans were carried out with an Eminence SET-3000GCT-X scanner (Shimadzu Corp, Kyoto, Japan) to measure regional brain radioactivity. No arterial blood sampling or metabolite analysis was performed. This scanner provides 99 sections with an axial field of view (FOV) of 256 mm. The in-plane and axial spatial resolutions were 3.45 mm FWHM and 3.72 mm FWHM, respectively. A head fixation device was used during the scans. A 10-min transmission scan using a ^137^Cs point source was performed to correct for attenuation. A dynamic PET scan was performed for 60 min (20 s × 9, 1 min × 5, 2 min × 4, 4 min × 11) after intravenous bolus injection of ^18^F-FE-PE2I. The injected radioactivity was 175.0 to 194.0 (185.5 ± 4.2) MBq. We used a filtered back-projection (FBP) image reconstruction algorithm with a second-order Gaussian filter with a cut off of 0.8 mm. Scatter correction was carried out with the hybrid dual-energy window (HDE) method. Motion correction was not used in this study.

### MRI Procedures

A 1.5 T magnetic resonance (MR) scanner (Intera 1.5 T Achieva Nova, Philips Medical Systems, Best, Netherlands) was used to acquire a high-resolution 3D fast spoiled gradient echo T1WI sequence (180 slices, 1 mm thickness, TR = 9.3 ms, TE = 4.6 ms, flip angle 10°, field of view 25 × 25 cm). The images were used as a reference for drawing volumes of interest (VOIs) on SPECT or PET images.

### Image Analysis

One neuroradiologist (T.S.) confirmed that no brain abnormalities were present in the subjects. DAT-SPECT uptake was semi-quantified based on three methods: the multi atlas-based method using MRI, single atlas-based method using SPECT and Bolt method. In the first two methods, uptake ratios were calculated for DAT-SPECT in the striatum, caudate, and putamen. In the latter method, the specific-to-non-displaceable binding ratio (SBR) was measured as a substitute for specific uptake ratios. The uptake ratios were calculated for DAT-PET in the striatum, caudate, and putamen in a standard manner based on the multi atlas-based method using MRI (17). For subsequent analyses, the quantified values for DAT-PET were defined as the reference values. The details of the subsequent analyses are described next.

### DAT-SPECT Images Analysis

#### Multi-Atlas-Based Method Using MRI (PNEURO)

The PNEURO tool (version 4.0, PMOD Technologies Ltd., Zurich, Switzerland) was used for the entire process. The whole processes described below can be performed semi-automatically, without any user interaction. It took ~10 min to perform the analysis for each case. First, rigid transformation between DAT-SPECT and 3D-T1WI was performed. Then, automatic segmentation was performed using subject-specific T1 images, creating VOIs for each of the striatum and caudate, putamen, and an outline of the cerebellum, occipital cortex and whole brain (excluding the striatum, ventricles and cerebellum) as a reference in each individual participant. To set these VOIs, we used the 1 mm Hammers atlas-N30R83 maximum probability atlas. The atlas map comprised gray matter as determined from segmentation of the subjects' T1 images into 30 bilateral cortical areas (including the amygdala and hippocampus), five bilateral deep nuclei (caudate, putamen, ventral striatum, thalamus, and pallidum), the bilateral cerebellum and the brainstem. This was accomplished in PNEURO using a pre-defined database of eight normal T1 MRI scans, each of which was manually segmented by neuroanatomically trained operators, with the most comparable brain hemispheres to those in a specific subject's T1 images selected using anatomical landmarks (anterior/posterior commissure, inter-hemispheric point, and inter-caudate point) and a calculation of the average thickness of the frontal horn of the left and right ventricles (12). The selected knowledge-based hemispheres were then elastically matched to subject hemispheres using a hierarchical approach [(1) global affine transformation, (2) individual structure adjustment with a free-form deformation algorithm] to create a set of structure definitions that was combined with the gray and white matter segmentation to produce a maximum probability atlas of base structures (deep nuclei, gray matter, cerebellum), with the gray matter (at probability>0.3) being further parcellated *via* intersection with the specified cortical atlas (here, Hammers). After parcellation, the VOIs were warped to SPECT images. SPECT uptake values were measured using MR-based anatomical VOIs to limit the SPECT-active volume in a reproducible manner. VOI-based analysis was then performed to extract specific target uptakes in the striatum, caudate, and putamen. To calculate uptake ratios, we chose the following three regions as a reference region: the cerebellum, occipital cortex and whole brain (excluding deep nuclei, the brainstem, the cerebellum and ventricles). As a result, the uptake ratio of the striatum (SUR), uptake ratio of the putamen (PUR) and uptake ratio of the caudate (CUR) were calculated based on each of the three reference regions. In addition, we also measured the volumes of the striatum, caudate, and putamen; their volumes were calculated by summing the right and left sides. Each semi-quantitative uptake ratio was calculated using the following formula: semi-quantitative uptake ratio = (mean counts in the target VOI)/(mean counts in the reference VOI).

**Figure 1:**
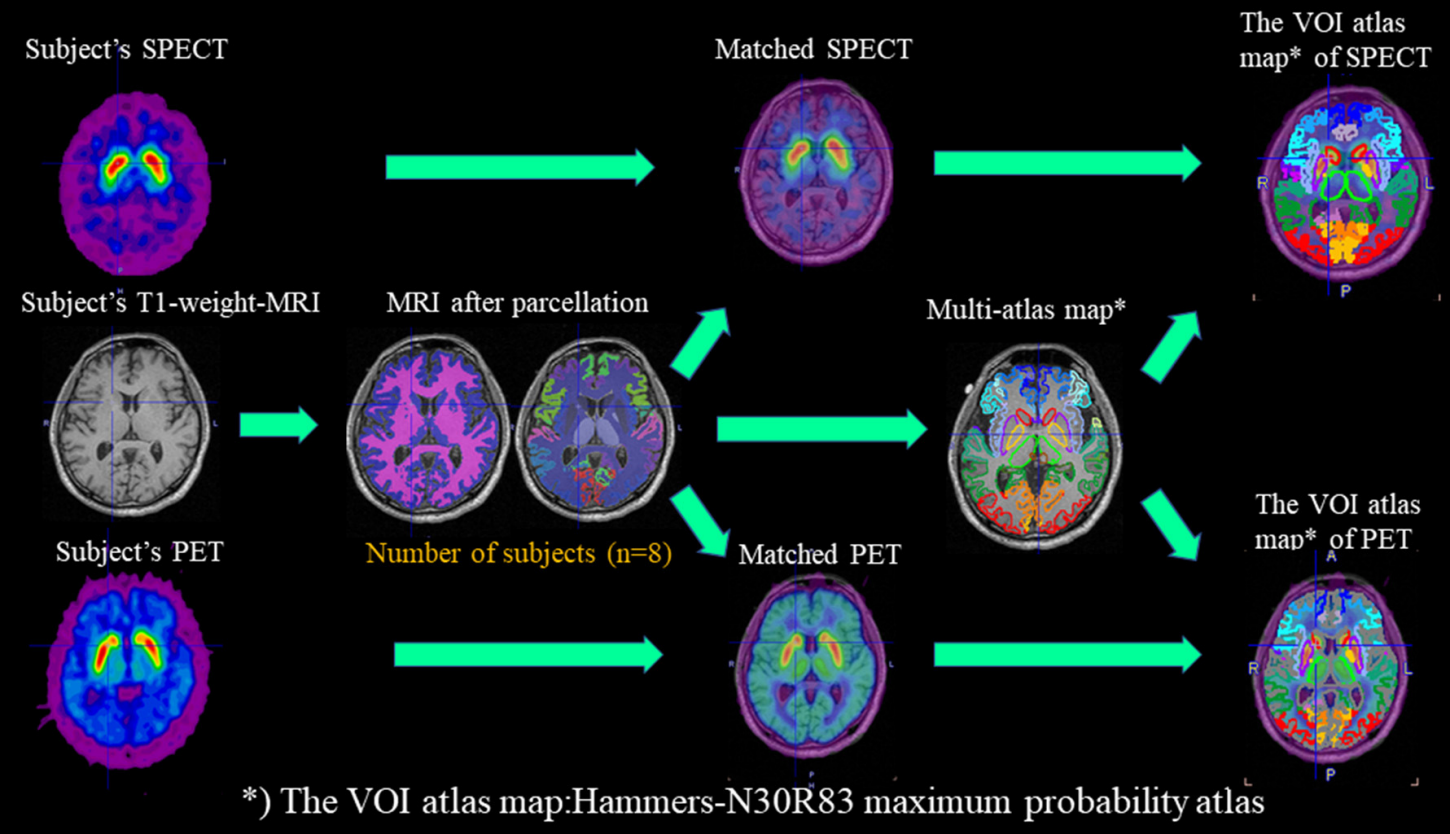
Workflow for studies with DAT-SPECT/PET and T1-MRI in PNEURO. In the workflow, subject-specific images (DAT-PET and DAT-SPECT) and MRI images after parcellation with eight normal subjects are matched, and multi-atlas maps are generated.

The workflow for studies with DAT-SPECT/PET and T1-MRI in PNEURO is shown in the upper part of Figure 1.

#### Single Atlas-Based Method Using SPECT (DaTQUANT)

DaTQUANT was used for the entire process (7). After SPECT reconstruction, the transaxial slices were used as input for the software. Non-rigid registration was applied to patient-specific SPECT data to match predefined SPECT-atlas data. Predefined template VOIs on the SPECT atlas were automatically positioned in the target regions, including the striatum, caudate, and putamen. The occipital cortex was also segmented as a reference region. The program calculated the SUR, CUR, and PUR as the ratio of each target region to the reference region.

### DaTView (DAT-SPECT Analysis)

We used DaTView (Aze, Tokyo, Japan) for the entire process. The procedure was the same as the method proposed by Tossici-Bolt et al. (5). DaTView applies the whole brain, except a region around the basal ganglia, as a reference region. SBR was defined as

$$\begin{array}{r} \text{SBR}=\text{Cs}/\text{Cr}, \end{array}$$

where Cs is the count concentration in the striatum due to specific binding only and Cr is the count concentration in the reference region due to non-specific binding. SBR is calculated from a sufficiently large VOI, including all counts associated with striatal activity, to be independent from the size of the VOI and from the resolution of the SPECT system. In order to avoid extrastriatal heterogeneous tissue counts, an average striatum volume of 11.2 mL was applied. In addition, we also used the cerebrospinal fluid correction (CSF-c) developed by Mizumura and equipped in DaTView to calculate the SBR (23).

### DAT-PET Images Analysis

We applied a simplified reference tissue model (14, 19, 24). To obtain semi-quantitative measures of SUR, CUR, and PUR for DAT-PET, static images were created by summing the dynamic scans between 32 and 60 min. The VOIs were created on these summed images with PNEURO. As in the the method used for the PNEURO DAT-SPECT analysis, after parcellation, the MRI and PET data were matched. PET activity values were applied to the MR-based anatomical VOIs to limit the PET-active volume in a reproducible manner. We also calculated 3 sets of semi-quantitative values with the cerebellum as a reference region (14, 17). The workflow for studies with DAT-PET and T1-MRI in PNEURO is shown in the lower part of Figure 1.

### Statistical Analysis

To validate the accuracy of DAT-SPECT quantification, the three types of correlations were statistically tested. First, the correlation of age with DAT-SPECT activity in each of the three methods was calculated with Pearson's correlation coefficient. Second, the correlation between DAT-SPECT and the reference, DAT-PET, in each region (e.g., the striatum, caudate, and putamen) was calculated with Pearson's correlation coefficient. Third, the correlation between region volume and age and that between region volume and DAT-SPECT activity in each of the three methods were calculated with Pearson's correlation coefficient. All statistical analyses were performed using IBM SPSS Statistics (Version 25.0, IBM Corp, Armonk, NY, USA).

## Results

**Figure 2:**
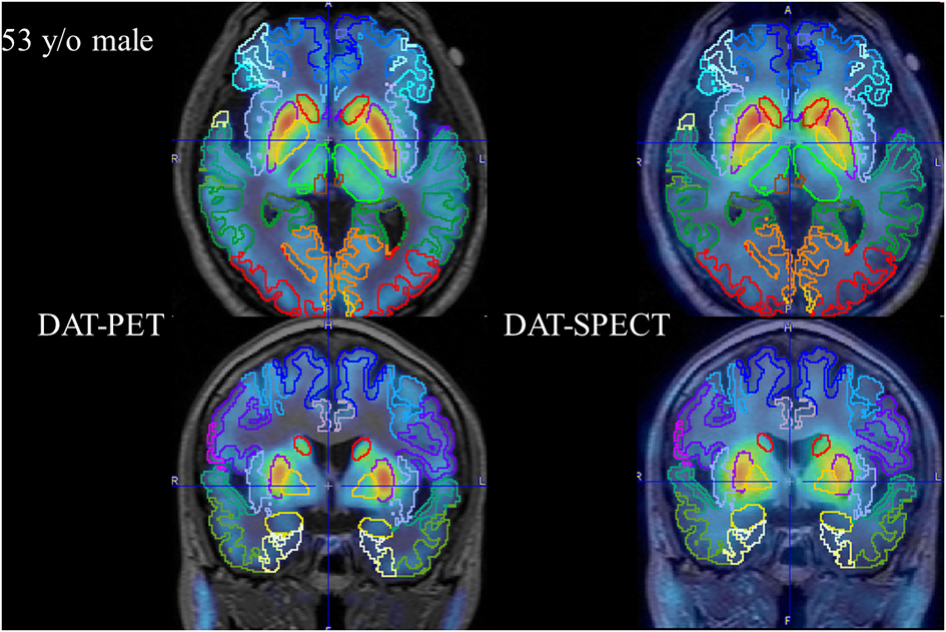
Representative case (53-year-old male). Axial and coronal fused PET (left column) and fused SPECT (right column) are shown.

The PNEURO analysis after parcellation successfully matched MRI and SPECT/PET data for each of the 30 cases. A representative case (53-year-old male) is shown in Figure 2.

**Figure 3:**
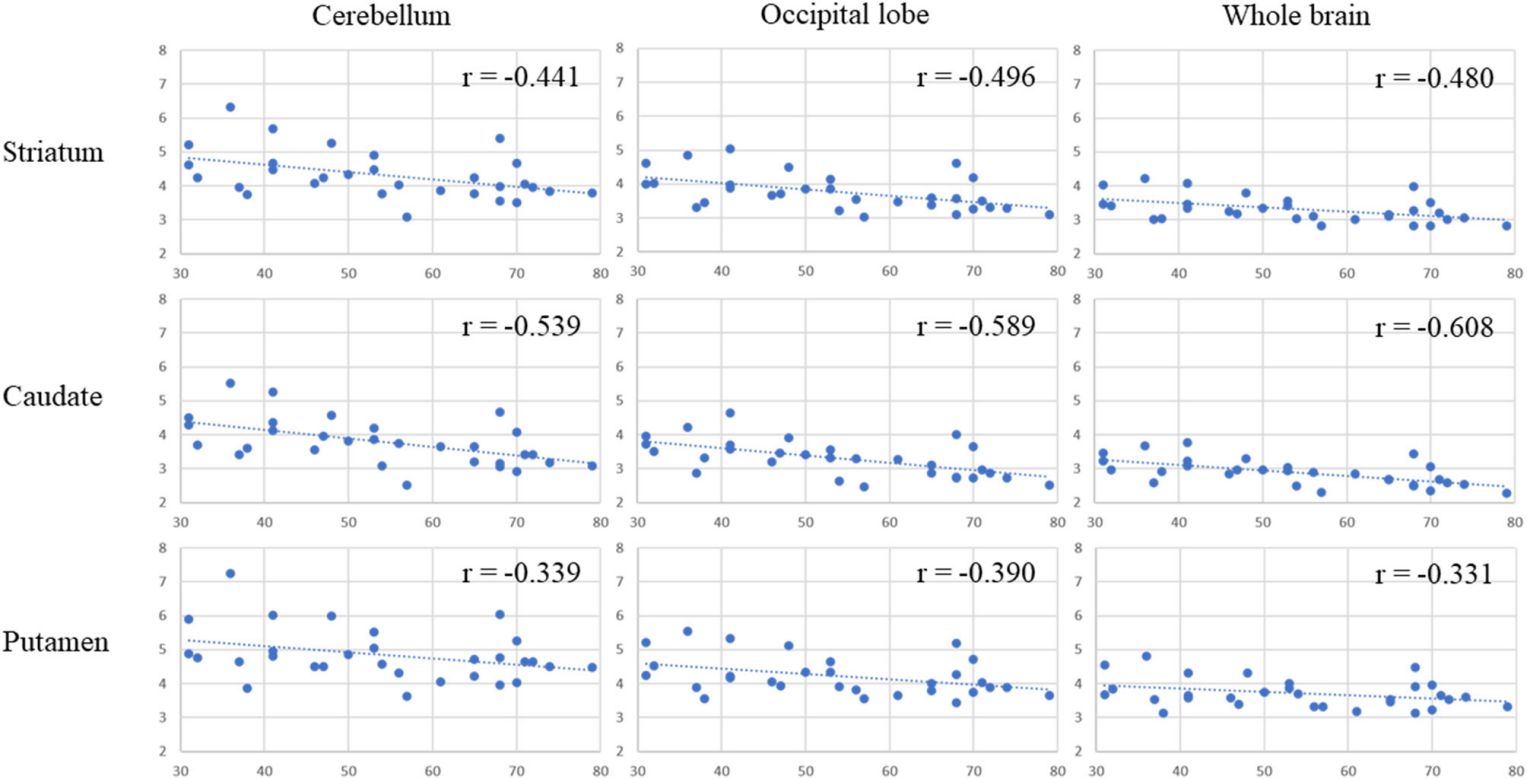
Age correlation with DAT activity, measured with DAT-SPECT and DAT-PET in the multi-atlas MRI-based method. In each correlation figure, the vertical axis indicates the semi-quantitative uptake for DAT-SPECT measured by the multi-atlas MRI-based method, and the horizontal axis indicates age. There are 3 × 3 correlation figure patterns in two different cases: where the target region is the striatum, caudate, or putamen and where the reference area is the cerebellum, occipital lobe or whole brain.

**Figure 4:**
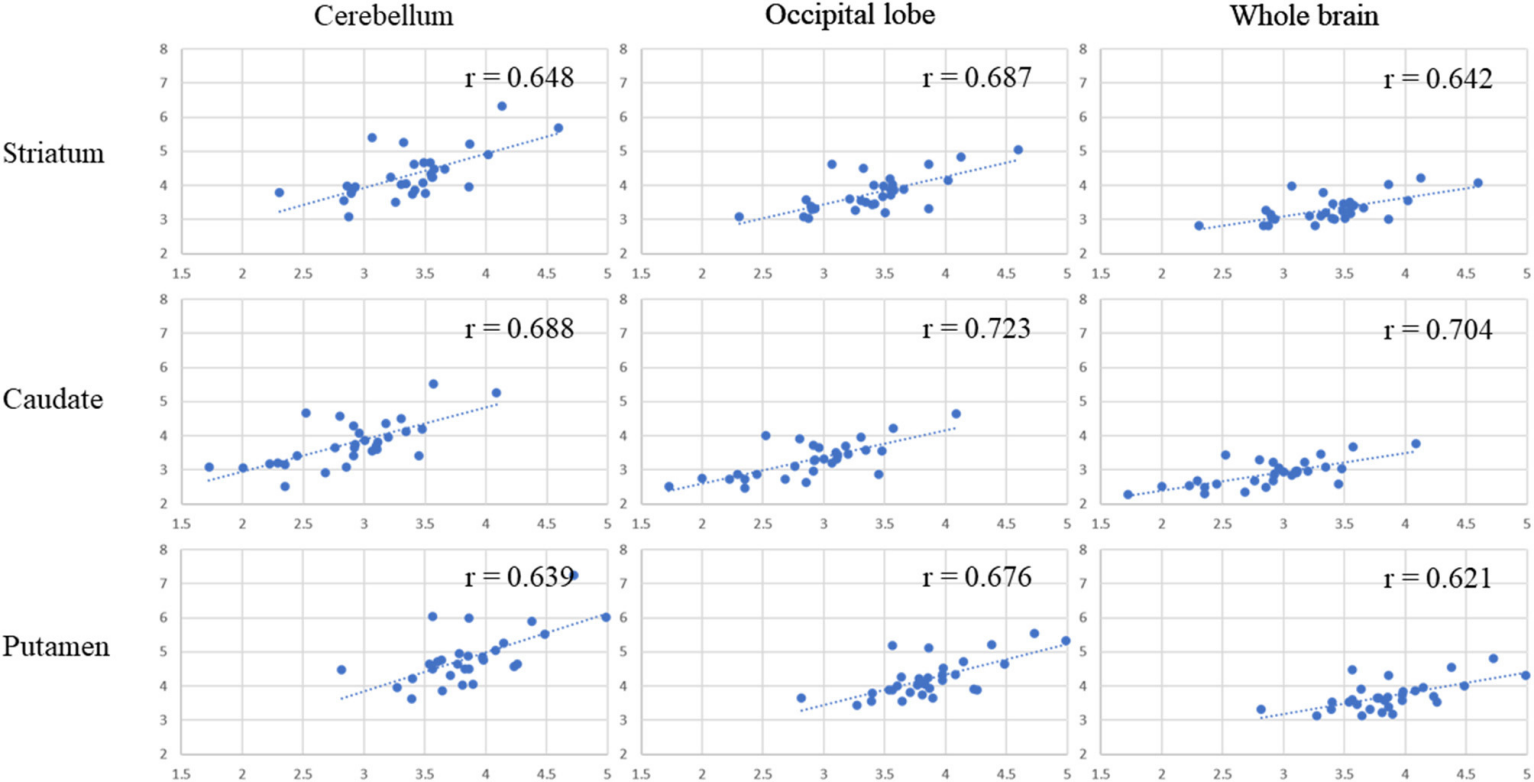
Correlation between the SUR, CUR, and PUR of DAT-SPECT and those of DAT-PET in the multi-atlas MRI-based method. In each correlation figure, the vertical axis indicates the semi-quantitative uptake for DAT-SPECT, and the horizontal axis indicates the semi-quantitative uptake for DAT-PET. There are 3 × 3 correlation figure patterns in two different cases: where the target region is the striatum, caudate, or putamen and where the reference area is the cerebellum, occipital lobe or whole brain.

Table 1 presents the results for the mean SUR/SBR, CUR and PUR from the DAT-SPECT and DAT-PET analyses. The correlation coefficients (*r* values) between age and SUR/SBR, CUR and PUR for DAT-SPECT and DAT-PET are presented in Table 2A; those between age and SUR, CUR and PUR for DAT-SPECT and DAT-PET in the PNEURO analyses are shown in Figure 3. Significant correlation between age and each semi-quantitative value was observed, except for SPECT-PUR, in the PNEURO analyses using the cerebellum or whole brain as the reference region. The annual decline rate of DAT activity with age was −0.232 to −0.691% depending on the measurement method (Table 2B). The *r* values for SUR/SBR, CUR and PUR between DAT-SPECT and DAT-PET are presented in Table 3; those between DAT-SPECT and DAT-PET in the PNEURO analyses are shown in Figure 4. All three types of SPECT semi-quantified values showed significant correlation to the values of PET (*r* = 0.616–0.723). In the PNEURO analyses, there was a tendency toward higher correlation when using the occipital lobe as the reference region than when using other regions (i.e., the cerebellum or whole brain). Regarding semi-quantification in the detailed regions (i.e., CUR or PUR), PNEURO using the occipital lobe as the reference region showed higher correlation than DaTQUANT (CUR 0.723 vs. 0.660, PUR 0.676 vs. 0.616). Table 4A shows the results for the mean volumes of the striatum, caudate and putamen. The *r* values indicated higher correlation of the volumes of the striatum, occipital lobe, cerebellum and whole brain to age (*r* = −0.519 to −0.678; Table 4B). The percentage volume decrease in these regions was −0.393 to −0.483% (Table 4C). The *r* values between volume and SUR/SBR, CUR and PUR are presented in Table 4D. The table shows that only one value in the DaTQUANT analysis, PUR, depended on volume, but only slightly (*r* = 0.383, *p* = 0.037).

**Table 1:** The SUR/SBR, CUR, and PUR of DAT-SPECT and DAT-PET.

**Radiopharmaceutical**	**Analysis tool**	**Reference area**	**CSF-C**	**SUR/SBR**		**CUR**		**PUR**	
				**Mean**	**SD**	**Mean**	**SD**	**Mean**	**SD**
DAT-SPECT	PNEURO	Cerebellum	–	4.323	0.706	3.783	0.683	4.843	0.779
		Occipital lobe	–	3.768	0.540	3.298	0.540	4.220	0.577
		Whole brain	–	3.308	0.388	2.831	1.102	3.708	0.433
	DaTQUANT	Occipital lobe	–	2.696	0.573	2.952	0.598	2.550	0.574
	DaTView	Whole brain	–	7.151	1.683	–	–	–	–
		Whole brain	+	6.259	1.487	–	–	–	–
DAT-PET	PNEURO	Cerebellum	–	3.391	0.454	2.888	0.499	3.868	0.436

*SUR, striatum uptake ratio; SBR, specific binding ratio; CUR, caudate uptake ratio; PUR, putamen uptake ratio; CSF-C, cerebrospinal fluid correction*.

**Table 2A:** Age correlation with DAT activity, measured with DAT-SPECT and DAT-PET.

**Radiopharmaceutical**	**Analysis tool**	**Reference area**	**CSF-C**	**SUR/SBR**		**CUR**		**PUR**	
				***r***	***p***	***r***	***p***	***r***	***p***
DAT-SPECT	PNEURO	Cerebellum	–	−0.441	0.015	−0.539	0.002	−0.339	0.067
		Occipital lobe	–	−0.496	0.005	−0.589	0.001	−0.390	0.033
		Whole brain	–	−0.480	0.007	−0.608	<0.001	−0.331	0.074
	DaTQUANT	Occipital lobe	–	−0.488	0.006	−0.483	0.007	−0.481	0.007
	DaTView	Whole brain	–	−0.665	<0.001				
		Whole brain	+	−0.635	<0.001				
DAT-PET	PNEURO	Cerebellum	–	−0.701	<0.001	−0.743	<0.001	−0.601	<0.001

*SUR, striatum uptake ratio; SBR, specific binding ratio; CUR, caudate uptake ratio; PUR, putamen uptake ratio; CSF-C, cerebrospinal fluid correction*.

**Table 2B:** Annual decline rate of DAT activity for Age, measured with DAT-SPECT and DAT-PET.

**Radiopharmaceutical**	**Analysis tool**	**Reference area**	**CSF-C**	**SUR/SBR**	**CUR**	**PUR**
DAT-SPECT	PNEURO	Cerebellum	–	−0.391	−0.491	−0.312
		Occipital lobe	–	−0.386	−0.488	−0.306
		Whole brain	–	−0.320	−0.432	−0.232
	DaTQUANT	Occipital lobe	–	−0.515	−0.494	−0.531
	DaTView	Whole brain	–	−0.680		
		Whole brain	+	−0.691		
DAT-PET	PNEURO	Cerebellum	–	−0.479	−0.598	−0.372

*SUR, striatum uptake ratio; SBR, specific binding ratio; CUR, caudate uptake ratio; PUR, putamen uptake ratio; CSF-C, cerebrospinal fluid correction*.

**Table 3:** Correlation between the SUR/SBR, CUR and PUR of DAT-SPECT and those of DAT-PET.

**Analysis tool**	**Reference area**	**CSF-C**	**SUR/SBR**		**CUR**		**PUR**	
			***r***	***p***	***r***	***p***	***r***	***p***
PNEURO	Cerebellum	–	0.648	<0.001	0.688	<0.001	0.639	<0.001
	Occipital lobe	–	0.687	<0.001	0.723	<0.001	0.676	<0.001
	Whole brain	–	0.642	<0.001	0.704	<0.001	0.621	<0.001
DaTQUANT	Occipital lobe	–	0.698	<0.001	0.660	<0.001	0.616	<0.001
DaTView	Whole brain	–	0.655	<0.001				
	Whole brain	+	0.659	<0.001				

*UR, striatum uptake ratio; SBR, specific binding ratio; CUR, caudate uptake ratio; PUR, putamen uptake ratio; CSF-C, cerebrospinal fluid correction*.

**Table 4A:** Volume (mL) of striatum, caudate, and putamen.

**Striatum**		**Caudate**		**Putamen**	
**Mean**	**SD**	**Mean**	**SD**	**Mean**	**SD**
20.941	2.873	8.691	1.449	12.250	1.582

**Table 4B:** Correlation between volume and age.

	***r***	***p***
Striatum	−0.529	0.003
Caudate	−0.367	0.046
Putamen	−0.342	0.064
Occipital lobe	−0.519	0.003
Cerebellum	−0.678	<0.001
Whole brain	−0.567	0.001

**Table 4C:** Annual decline rate (%) of volume for age.

Striatum	−0.393
Caudate	−0.343
Putamen	−0.261
Occipital lobe	−0.416
Cerebellum	−0.483
Whole brain	−0.425

**Table 4D:** Correlation between Volume and the SUR/SBR, CUR, and PUR of DAT-SPECT and DAT-PET.

**Radiopharmaceutical**	**Analysis tool**	**Reference area**	**CSF-C**	**SUR/SBR**		**CUR**		**PUR**	
				***r***	***p***	***r***	***p***	***r***	***p***
DAT-SPECT	PNUERO	Cerebellum	–	−0.014	0.942	0.175	0.354	−0.128	0.501
		Occipital lobe	–	−0.013	0.945	0.166	0.381	−0.136	0.475
		Whole_brain	–	0.021	0.913	0.130	0.494	−0.061	0.750
	DaTQUANT	Occipital lobe	–	−0.190	0.315	0.225	0.232	−0.383	0.037
	DaTView	Whole_brain	–	0.081	0.672				
		Whole_brain	+	0.108	0.569				
DAT-PET	PNEURO	Cerebellum	–	0.167	0.377	0.370	0.044	0.008	0.967

*SUR indicates striatum uptake ratio; SBR, specific binding ratio; CUR, caudate uptake ratio; PUR, putamen uptake ratio; CSF-C, cerebrospinal fluid correction*.

## Discussion

In the current study, DAT-SPECT semi-quantification based on multi-atlas MRI-based methods showed comparable to higher correlation to age or DAT-PET values compared with clinically available methods. In multi-atlas-based MRI analyses, the occipital lobe can be used as a reference for more precise semi-quantification than the cerebellum or whole brain. To our knowledge, this is the first study to validate the performance of semi-automatic multi-atlas MRI-based parcellation for DAT-SPECT. Furthermore, in regard to the comparisons between DAT-SPECT and DAT-PET, the current study recruited more age-generalized healthy controls (i.e., a uniform distribution of younger to older ages) than any of the previous studies.

### Correlation Between Age and DAT-SPECT

Significant correlation was observed between age and all types of semi-quantitative value for DAT-SPECT. The correlation coefficients (*r* values) in this study showed a tendency to be equal to those in previous studies. In this study, the *r* values between age and SUR/SBR, CUR, and PUR for DAT-SPECT were −0.441 to −0.665, −0483 to −0.608, and −0.331 to −0.481, respectively. In previous studies, the *r* values between age and SUR/SBR, CUR, and PUR for DAT-SPECT were −0.449 to −0.632, −0.496 and −0.400, respectively (18, 19, 21, 25, 26). The *r* values between age and SBR based on the Bolt method were relatively higher than those based on the other methods (−0.635 to −0.665 vs. −0.441 to −0.496). It is assumed that the SBR method overestimates the reduction with age because it does not take the age-dependent decline in striatal volume into account (21). We revealed that the striatal volume declined annually (0.393% per year) despite the fact that the Bolt method defines a fixed striatum volume (11.2 mL) (5). DaTQUANT is generally considered to also be vulnerable to atrophy of the target regions. This method transforms the subject's specific target tracer accumulation into a SPECT template. As a result, it could overestimate the decrease in DAT activity in patients because the putamen volume in Parkinson's disease and multiple system atrophy has a tendency to decrease (27, 28). Therefore, MRI-based VOI delineation is necessary for *in vivo* DAT quantification.

### Correlation Between DAT-PET and DAT-SPECT

DAT-PET has been shown to be an excellent imaging tool for *in vivo* DAT quantification in the entire nigrostriatal tract (13, 14, 20). There are also some studies concerning the performance of DAT-PET for *in vivo* DAT quantification and comparisons of diagnostic value between normal subjects and those with Parkinson's disease/Parkinsonism (11, 17, 18). One of the expected merits of DAT-PET with higher spatial resolution is the segmentation of tracer accumulation into caudate and putamen regions. A detailed evaluation based on this segementation enables differentiation of neurodegenerative Parkinsonism (29, 30). However, in clinical practice, the use of DAT-PET entails higher costs and higher radiation exposure. It is necessary to enhance the utility of DAT-SPECT semi-quantification by combining several methodological approaches, such as accurate VOI delineation and the selection of appropriate reference tissue.

Regarding SUR, the correlation coefficient between the semi-quantitative values for DAT-SPECT and DAT-PET did not significantly differ among the three methods. The multi-atlas MRI-based method is at least comparable to clinical methods for the semi-quantification of this region. Regarding CUR and PUR, in particular, the method showed higher correlation than the SPECT-atlas method (0.723 and 0.676 vs. 0.660 and 0.616). The multi-atlas MRI-based method is expected to be utilized to detect subtle changes of DAT activity in the caudate and putamen, which may impact the diagnosis of Parkinson's disease/syndrome. This expectation is also supported by previous studies (17, 18).

### Selection of the Reference Region in the Multi-Atlas MRI-Based Method

In the current study, we sought to determine the appropriate reference regions for accurate DAT-SPECT semi-quantification. The occipital cortex proved to be the best region, with stronger correlation to age and DAT-PET compared with the cerebellum or whole brain. In a study with a similar concept to that of the current study, Delva et al. investigated the location of the optimal reference tissues for DAT imaging (17). They recruited nine patients with early Parkinson's disease and 34 healthy volunteers. All participants underwent DAT-PET with simultaneous acquisition of MRI, which was further used for VOI delineation. The results showed that the occipital cortex may be preferable as the reference region compared with the cerebellum, which supports the results of the current study. Another of their studies also supported the results of the current study (17). It should be noted, however, that our result cannot be translated directly into clinical scans. In our study, only HVs were recruited. In cases with dementia (e.g., dementia with Lewy bodies and Alzheimer's disease), several brain morphological changes can be present (31–34). Even when complicated morphological changes occur, the multi-atlas MRI-based approach would be useful thanks to its flexibility with respect to multiple outputs. The pre-processing performed for accurate delineation of each brain region enables mapping of the output of the multiple semi-quantification results based on each reference region. In addition, the degree of the atrophy (net volume) in each corresponding target or reference region would be easily obtainable.

### Limitations

The current study has some limitations. First, it is known that ^18^F-FE-PE2I is a more selective ligand to DAT than ^123^I-FP-CIT. There is concern regarding the difference between the pharmacokinetics of ^123^I-FP-CIT and that of ^18^F-FE-PE2I. However, previous studies supported high correlation for both of these ligands (17, 18). Second, we applied one of the standard co-registration methods, the mutual information matching algorithm, provided by a single tool (PNEURO) (35–37). Other co-registration algorithms, such as MRtrix and ANTs, may improve or lead to different results (38–41). Further investigations utilizing multiple pipelines should be conducted. Third, the clinical impact of the multi-atlas MRI-based method was not clarified, because we analyzed only normal volunteers and did not assess any subjects with Parkinson's disease or Parkinsonism. We used the correlation to age and to DAT-PET value as surrogate indices to validate the accuracy of the current method, and this method has been widely accepted in this kind of study (7, 8, 17–19, 21, 25, 26). The multi-atlas MRI-based method is expected to be more useful in disease conditions because it compensates for the difficulty in segmentation in patients with morphological changes or decreased uptake, but further studies should be conducted. Fourth, a PNEURO analysis takes relatively more time (i.e., ~10 min), though the whole process is performed semi-automatically. This issue will undoubtedly be resolved when computer technology gets better. Fifth, the attenuation correction in DAT-PET was different from that in DAT-SPECT, which might have impacted the correlation between the two tracers. This is an inherent limitation of a comparison study between DAT-PET and DAT-SPECT (17, 18). Sixth, we performed DAT-PET quantification with a simplified reference tissue model without motion correction, partial volume correction or dynamic data analysis (14, 19). We tried to maintain the uniformity of the methodology between DAT-PET and DAT-SPECT analysis. The accuracy of the DAT-PET data was partially validated in the current study, as there was sufficient age correlation of DAT-PET data (*r* = −0.601 to −0.743) compared with previous studies (18, 19).

## Conclusion

Multi-atlas MRI-based parcellation for DAT-SPECT semi-quantification is at least comparable to the current clinical methods in terms of the correlation to age and to DAT-PET quantification. In this method, the occipital lobe is the best region to use as the reference. The method is expected to provide detailed and robust semi-quantification in the putamen and caudate nucleus regardless of abnormal brain shape or atrophy of brain tissue.

## Data Availability Statement

The raw data supporting the conclusions of this article will be made available by the authors, without undue reservation.

## Ethics Statement

The studies involving human participants were reviewed and approved by Committee on Clinical Practice of Investigational New Drugs, Nippon Medical School. The patients/participants provided their written informed consent to participate in this study. Written informed consent was obtained from the individual(s) for the publication of any potentially identifiable images or data included in this article.

## Author Contributions

All authors listed have made a substantial, direct and intellectual contribution to the work, and approved it for publication.

## Conflict of Interest

The authors declare that the research was conducted in the absence of any commercial or financial relationships that could be construed as a potential conflict of interest.
